# Composite dietary antioxidant index was negatively associated with the prevalence of diabetes independent of cardiovascular diseases

**DOI:** 10.1186/s13098-023-01150-6

**Published:** 2023-09-08

**Authors:** Xiaojie Chen, He Lu, Yingwei Chen, Haiqiang Sang, Yi Tang, Yifan Zhao

**Affiliations:** 1 Department Cardiology, The First Affiliated Hospital of Zhengzhou University, Zhengzhou, China; 2 Department of Cardiology, The People’s Hospital of Jiawang District of Xuzhou, XuZhou, China

**Keywords:** Composite dietary antioxidant index, Diabetes, Antioxidant, NHANES, Cross-sectional study

## Abstract

**Aim:**

The association between composite dietary antioxidant index (CDAI) and diabetes remains unknown. Our study was to investigate the association of CDAI with diabetes.

**Methods:**

A total of 11,956 participants were enrolled from the National Health and Nutrition Examination Surveys (NHANES). The CDAI was calculated from the intake of six dietary antioxidants. Multivariable logistic regressions were performed to explore the associations between CDAI and the prevalence of diabetes and glycemic index. Non-linear associations were explored using restricted cubic splines.

**Results:**

In the multivariate logistic regression model, the odds ratio (95% confidence interval) of CDAI associating with obesity was 0.98 (0.97-1.00; p = 0.033). Compared with the lowest quartile, the highest quartile was related to 0.84-fold risk of diabetes (0.71–0.99; p = 0.035). However, CDAI was not independently associated with fasting glucose and hemoglobin A1c.

**Conclusion:**

CDAI was negatively associated with diabetes and the relationship was independent of other traditional risk factors.

## Introduction

Diabetes is a group of metabolic diseases, defined by as hyperglycemia caused by the defect of insulin secretion and action [1].The prevalence of diabetes has increased over the past several decades, with Type 2 diabetes making about 90% of the cases, which accounts for over 430 million people worldwide [2]. Diabetes is often associated with significant organ damage and failure, which leads to an increase in mortality rates. Almost all diabetes-related complications can be attributed to vascular damage, including macrovascular complications and microvascular complications [3]. Thus, investigating more risk factors and effective interventions for diabetic individuals is extremely urgent.

It is reported that dietary habit is also an important trigger of diabetes [4]. Previous studies have investigated the relationship between dietary antioxidants and diabetes [5, 6] and diabetic complications [7, 8]. Dietary total antioxidant capacity (TAC) as an indicator of diet quality, has been associated with insulin resistance and cardiometabolic risks [9]. However dietary TAC varies according to the geographic location, seasonality, water and sun availability, storage conditions, food processing, and cooking of the examined food group. The Composite Dietary Antioxidant Index (CDAI) is a valid and reliable nutritional tool to assess overall antioxidant characteristics of the diet, which is a summary score of six dietary antioxidants including vitamins A, C, and E, manganese, selenium, and zinc [10]. Previous studies have found that CDAI was associated with depression [11] and colorectal cancer [12]. However, the investigation on the association between CDAI and diabetes has been scared.

Vitamin A participates in multiple metabolic processes and has an important effect on insulin sensitivity [13]. Its concentration was lower in type 2 diabetic patients [14]. It was reported that diabetic patients have increased lipid peroxidation and decreased Vitamins C and E [15]. Plasma Vitamin C was inversely correlated to glycosylated hemoglobin and blood glucose [16]. Manganese is an essential trace metal element and deficiency or excessive Manganese exposure could increase ROS generation and result in further oxidative stress [17]. A Chinese population-based study found a U-shaped association between manganese with diabetes [18]. Selenium functions metabolically as an essential constituent of selenoproteins, and has a link with diabetes risk [19]. Zinc appears to activate key molecule in cell signaling, involved in the homeostasis of glucose and insulin receptors. Abnormal Zn may also cause diabetes complications [20]. Considering the relevance of the components of CDAI and diabetes, we aimed to examine the potential association between CDAI and the prevalence of diabetes.

## Methods

### Study Population

The study included participants from the National Health and Nutrition Examination Survey (NHANES), a nationwide survey conducted by the National Center of Health Statistics (NCHS). The survey was designed to assess the health and nutritional status of the non-institutionalized US population by a stratified and multistage sampling design. We combined four cycles of survey with completed data on the intake of components of CDAI from 2008 to 2014 (n = 13,116). After excluding adult participants with missing data on glucose and HbA1c (n = 1160), a total of 11,956 individuals were included in our analyses. All participants provided written informed consent and the protocol was approved by the Ethics Review Board of National Center for Health Statistics (Protocol #2011-17).

### Exposure and outcomes

Each participant’s food and nutrient intake in the NHANES dataset was recorded via a 24-h dietary recall interview. The first dietary recall was conducted in person and then 3 to 10 days later via telephone. The Food and Nutrient Database for Dietary Studies of the United States Department of Agriculture was used to calculate the intake of antioxidants, micronutrients, and total energy[21]. Based on the questionnaire interview, we determined the intake of dietary supplements during the past month, including dosage, frequency, and duration of consumption[22].

Standardized questionnaires were administered in the home, followed by a detailed physical examination and blood specimens at a mobile examination center. Diabetes was defined as (1) self-report of a diagnosis by a physician or (2) HbA1c ≥ 6.5% or (3) fasting plasma glucose ≥ 126 mg/dL.

### Covariates

To assess the influence of potential confounding factors, we selected several important covariates, including gender, age, race, education level, physical activity, and smoking status, which were collected by using standardized questionnaires. Weight and height of each participant were obtained from the physical examinations. Multiple imputation was performed for missing values.

### Statistical analysis

Participants were separated into two groups based on CDAI quartiles. Baseline variables differences were tested by Student t test and Chi-Square tests. The association between CDAI and diabetes was explored with logistic regression models, while the association between CDAI and glucose and HbA1c were explored with linear regression models. The restricted cubic splines were performed to explore the nonlinear association. All statistical analyses were done in R software, version 3.6 and P < 0.05 was regarded as significant.

## Results

The baseline features of participants were summarized in Table 1. The individuals with larger CDAI quartile tend to male (p < 0.001), younger (p < 0.001), and less percentage of diabetes (p < 0.001).

**Table 1:** Characteristics of the study population according to CDAI quartiles

Variable	Q1 (n = 2991)	Q2 (n = 2984)	Q3 (n = 2991)	Q4 (n = 2990)	P-value
Male (%)	1079 (36.1)	1274 (42.7)	1560 (52.2)	1989 (66.5)	< 0.001
Age, years	43.4 ± 21.9	44.8 ± 21.4	43.8 ± 20.3	42.2 ± 18.9	< 0.001
Race (%)					< 0.001
Non-Hispanic white	1178 (39.4)	1253 (42.0)	1286 (43.0)	1367 (45.7)	
Non-Hispanic black	743 (24.8)	605 (20.3)	569 (19.0)	526 (17.6)	
Mexican American	467 (15.6)	502 (16.8)	533 (17.8)	515 (17.2)	
Others	603 (20.2)	624 (20.9)	603 (20.2)	582 (19.5)	
Education					< 0.001
Less than high school	1178 (39.4)	1253 (42.0)	1286 (43.0)	1367 (45.7)	
High school	743 (24.8)	605 (20.3)	569 (19.0)	526 (17.6)	
More than high school	467 (15.6)	502 (16.8)	533 (17.8)	515 (17.2)	
Activity (%)					< 0.001
Vigorous	1051 (35.1)	909 (30.5)	840 (28.1)	854 (28.6)	
Moderate	1220 (40.8)	1291 (43.3)	1252 (41.9)	1129 (37.8)	
Inactive	720 (24.1)	784 (26.3)	899 (30.1)	1007 (33.7)	
Smoker, %					< 0.001
Current	2015 (67.4)	2184 (73.2)	2272 (76.0)	2193 (73.3)	
Past	154 (5.1)	149 (5.0)	132 (4.4)	176 (5.9)	
Never	822 (27.5)	651 (21.8)	587 (19.6)	621 (20.8)	
BMI, kg/m2	28.3 ± 7.1	28.2 ± 6.9	28.1 ± 6.9	27.8 ± 6.9	0.074
Hypertension (%)	458 (15.3)	449 (15.0)	424 (14.2)	385 (12.9)	0.032
CHD (%)	118 (3.9)	109 (3.7)	96 (3.2)	81 (2.7)	0.046
Glucose, mg/dL	106.6 ± 32.9	105.4 ± 29.8	106.4 ± 34.8	105.7 ± 32.7	0.456
HbA1c (%)	5.68 ± 1.01	5.69 ± 0.96	5.68 ± 1.04	5.62 ± 1.02	0.027
Diabetes (%)	520 (17.4)	485 (16.3)	452 (15.1)	381 (12.7)	< 0.001

Data are presented as mean (SD) or n (%). BMI, body mass index; HBP, hypertension; CHD, coronary heart disease; HbA1c, hemoglobin A1c

Multivariable logistic regression models were constructed to examine the relationship between CDAI and diabetes (Table 2). In Model 1, the odds ratio (OR) and 95% confidence interval (CI) was 0.97 (0.95–0.98; P < 0.001), which indicated that the risk of obesity was reduced for every unit rise in CDAI. The relationship still existed in the model 2 (OR [95% CI]: 0.97 [0.96–0.98]; P < 0.001) and model 3 (OR [95% CI]: 0.98 [0.97-1.00]; P = 0.033). Compared with the lowest quartile, the highest quartile was significantly associated with diabetes (OR [95% CI]: 0.84 [0.71–0.99]; P = 0.035). However, no dependent associations between CDAI and glucose or HbA1c were found (Table 3).

**Table 2:** Association of composite dietary antioxidant index and diabetes

	Model 1		Model 2		Model 3	
	OR [95% CI]	P	OR [95% CI]	P	OR [95% CI]	P
Q1	Ref	**-**	Ref	**-**	Ref	**-**
Q2	0.92 [0.81, 1.06]	0.242	0.85 [0.73, 0.98]	0.028	0.92 [0.79, 1.07]	0.256
Q3	0.85 [0.74, 0.97]	0.017	0.82 [0.70, 0.95]	0.008	0.93 [0.80, 1.08]	0.350
Q4	0.69 [0.60, 0.80]	< 0.001	0.71 [0.61, 0.84]	< 0.001	0.84 [0.71, 0.99]	0.035
CDAI	0.97 [0.95, 0.98]	< 0.001	0.97 [0.96, 0.98]	< 0.001	0.98 [0.97, 1.00]	0.033

Model 1 was not adjusted

Model 2 was adjusted for age and gender

Model 3 was adjusted for age, gender, race, education, activity, smoking status, hypertension, and CHD. OR, odds ratio; CI, confidence interval; CHD, coronary heart diseases

**Table 3:** Association of composite dietary antioxidant index and glycemic index

	Model 1		Model 2		Model 3	
	β [95% CI]	P	β [95% CI]	P	β [95% CI]	P
**Glucose**						
Q1	Ref	-	Ref	-	Ref	-
Q2	-1.16 [-2.81, 0.50]	0.171	-2.13 [-3.72, -0.54]	0.009	-1.49 [-3.07, 0.09]	0.065
Q3	-0.13 [-1.79, 1.52]	0.874	-1.28 [-2.87, 0.32]	0.117	-0.25 [-1.85, 1.36]	0.764
Q4	-0.84 [-2.50, 0.81]	0.317	-2.20 [-3.82, -0.57]	0.008	-0.82 [-2.45, 0.82]	0.329
**HbA1c**						
Q1	Ref	-	Ref	-	Ref	-
Q2	0.00 [-0.05, 0.05]	0.914	-0.02 [-0.07, 0.02]	0.331	0.01 [-0.04, 0.05]	0.794
Q3	-0.01 [-0.06, 0.04]	0.816	-0.02 [-0.07, 0.02]	0.321	0.02 [-0.03, 0.07]	0.359
Q4	-0.06 [-0.12, -0.01]	0.012	-0.07 [-0.12, -0.02]	0.006	-0.00 [-0.05, 0.05]	0.870

Model 1 was not adjusted

Model 2 was adjusted for age and gender

Model 3 was adjusted for age, gender, race, education, activity, smoking status, hypertension, and CHD. OR, odds ratio; CI, confidence interval; CHD, coronary heart diseases

**Fig. 1:**
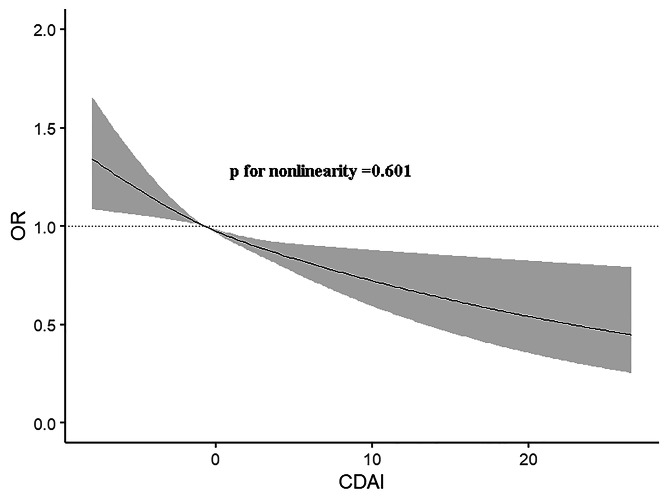
The dose-response relationship between CDAI and the prevalence of diabetes

Furthermore, restricted cubic spline suggested the relationship between CDAI and obesity was linear (P for nonlinearity = 0.601; Fig. 1).

## Discussion

In our study, we found that CDAI was negatively associated with diabetes. And the relationship remained even after adjusted other covariates, which indicated that CDAI was a protective factor for the development of diabetes. A dose-response analysis found that this negative relationship was linear.

A meta-analysis estimated antioxidant intake was associated with a 13% reduction of diabetes risk, mainly attributed to vitamin E and carotenoids. However, the reduction was not found to be substantial[23]. In addition to other fruits and vegetables contain antioxidant vitamins[24], some review demonstrated that the intake of magnesium [25], zinc [26] and selenium [27] are able to regulate inflammatory and oxidation cascade, which could mediate the effect of a healthy dietary pattern to diabetes. In consistent with these results, our study also found that CDAI, consisting of vitamins A, C, and E, manganese, selenium, and zinc, was inversely correlated with diabetes.

The mechanism is closely related to oxidative stress. Because multiple antioxidants may have synergistic effects. Many dietary antioxidants use their bioactive molecules to reduce oxidative stress and exert antioxidant effects [28]. So, antioxidant nutrients may be able to reduce the risk of diabetes caused by oxidative stress. However, the exact molecular mechanisms are not well-understood, and more research is needed.

There are several limitations to this study. Firstly, the diet assessment might involve measurement errors and inaccuracies. Secondly, bias is inevitable in cross-sectional studies.

## Conclusion

Our study found a negative association between CDAI and diabetes after adjusting for potential confounders.

## Data Availability

All data could be available upon request from the corresponding author.
